# A Rare Condition of Simultaneous Occurrence of Sigmoid and Transverse Colon Volvulus

**DOI:** 10.7759/cureus.20250

**Published:** 2021-12-07

**Authors:** Hossein Torabi, Kasra Shirini, Rona Ghaffari

**Affiliations:** 1 Department of General Surgery, Poursina Medical and Educational Center, Guilan University of Medical Sciences, Rasht, IRN; 2 Department of General Surgery, Iran University of Medical Science, Tehran, IRN; 3 Department of General Surgery, Poursina Medical and Educational Center, Guilan University of Medical Science, Rasht, IRN

**Keywords:** emergency condition, laparotomy, simultaneous occurrence, total colectomy, sigmoid, transvers colon, volvulus

## Abstract

Volvulus of the colon is the third important reason for colon obstruction, which can occur in different parts of the colon for various reasons and can lead to ischemia and necrosis of the colon wall tissue. In this article, we are going to describe a simultaneous sigmoid and colon volvulus which was operated on with suspicion of sigmoid volvulus. A 72-year-old patient presented with suddenly severe generalized abdominal pain with a past medical history of inflammatory bowel disease (IBD) and prolonged constipation who underwent laparotomy for suspected colonic volvulus. During the operation, it was found that transverse colon volvulus occurred simultaneously with sigmoid colon volvulus with colon necrosis along its length. Therefore, a total colectomy with ileorectal anastomosis was performed. After one week, the patient was discharged from the surgical ward after tolerating feeding and with stable vital signs. The simultaneous transverse colon and sigmoid volvulus is a rare phenomenon, and there are several ways to diagnose and evaluate this situation. However, none of them can help us diagnose this disease. Unfortunately, no specific algorithm has been designed for the approach in this situation, and it all depends on the patient’s condition. Simultaneous occurrence of the sigmoid and transverse colon can make a high-risk emergency condition that could threaten the patient’s life. Therefore, paying attention to the patient’s symptoms and patient’s condition and clinical findings, with high accuracy and speed and subsequently selecting the best surgical technique, if surgery is necessary, and according to the finding during surgery, especially the extent of necrotic tissue, the most crucial issue in treating the patient.

## Introduction

Tumor obstruction and complicated sigmoid diverticulitis are the most common reasons for colonic obstruction. After these incidents, colonic volvulus is the third cause of 3% to 5% of acute bowel obstruction [1-3]. Sometimes a part of the colon may twist around itself for a variety of reasons which is called colonic volvulus, and as a result of strangulation, it causes large bowel obstruction [2,3]. Therefore, it may lead to ischemia and then maybe followed by necrosis [2]. This problem is a common and serious health hazard in some areas such as Africa, Russia, and the Middle East. This disease is seen in different parts of the colon. The most common and significant parts are the sigmoid with a probability of 61%, followed by the cecum with a chance of 34.5%, and then the transverse colon with a possibility of 3.6% [1,4]. So, the simultaneous occurrence of sigmoid and transverse colon volvulus is a rare condition. This synchronization creates a particular emergent situation that must be treated as soon as possible, and if the disease is not diagnosed quickly and in the early stages, it can become a life-threatening condition [1-3]. The most important diagnostic ways are first the patient’s clinical presentation, such as no defecation, no gas-passing, and abdominal distension. Second, using imaging tools such as chest and abdominal X-rays and CT scans. The main and the most important way to treat and save the patient with dual simultaneous volvulus is surgery due to the possibility of tissue ischemia and necrosis [1-3]. In this report, an old man has presented with a synchronous sigmoid and transverse colon volvulus which occurs suddenly.

## Case presentation

**Figure 1:**
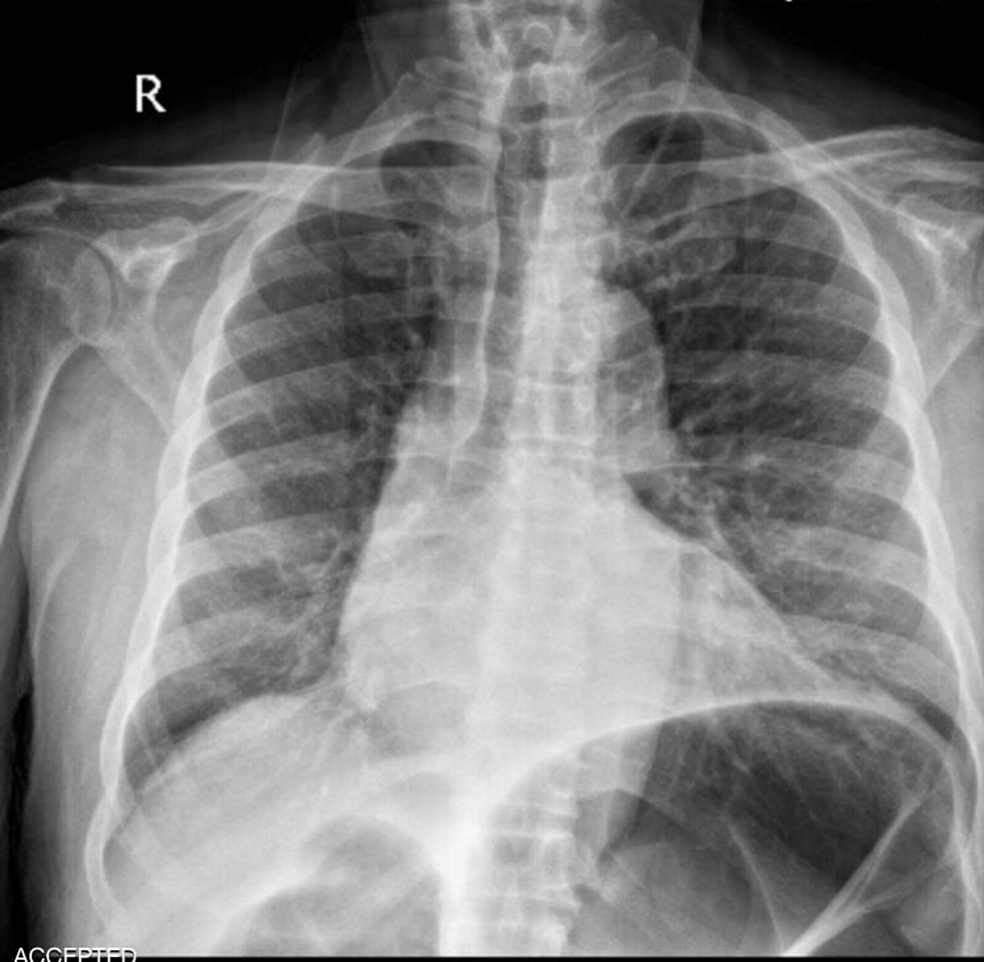
Upright chest X-ray

**Figure 2:**
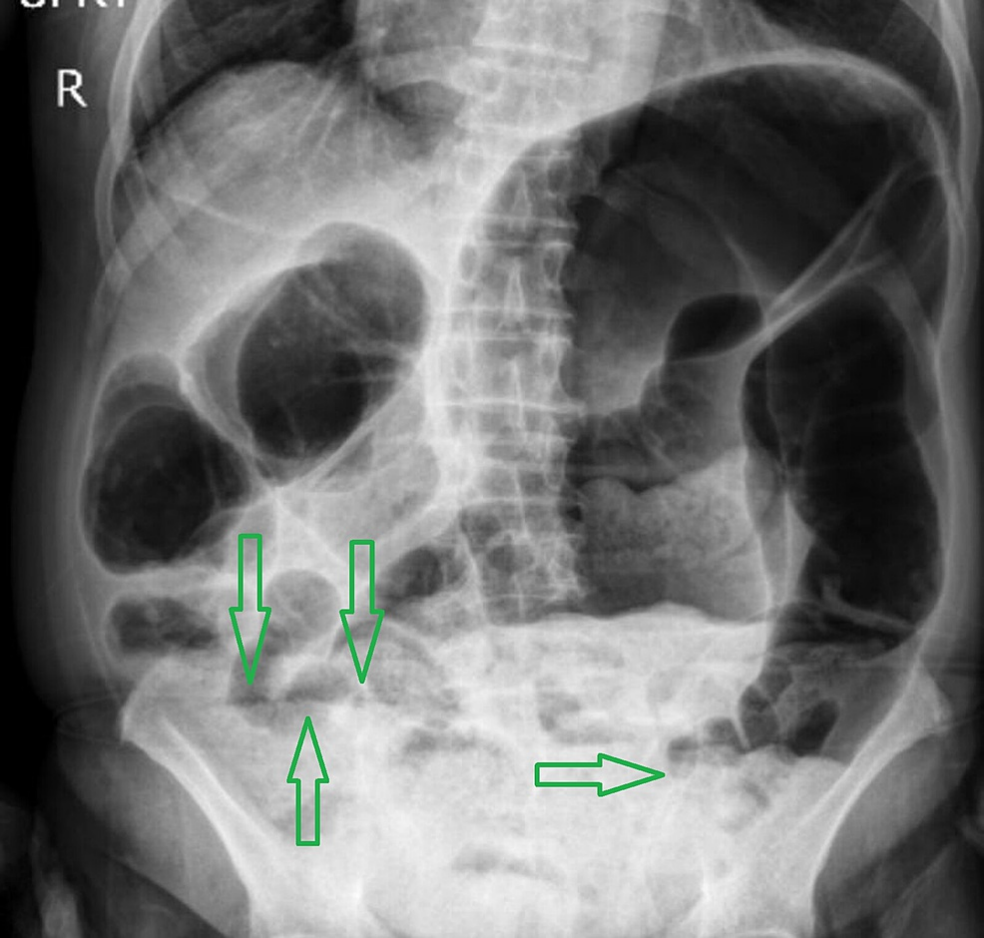
Upright abdominal X-ray Green arrows show air-fluid levels

**Figure 3:**
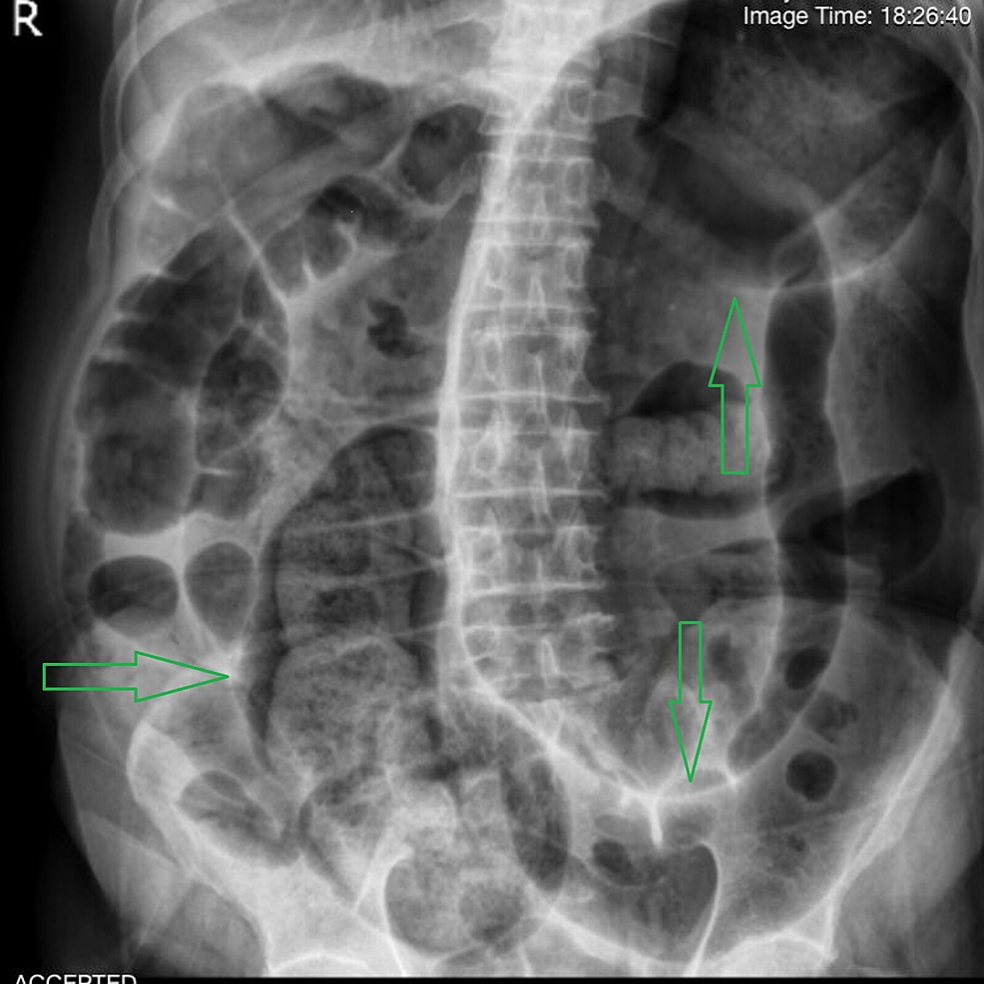
Supine abdominal X-ray Green arrows show dilated bowel loops

A 72-year-old male presented to the surgical emergency department with a four-day history of severe generalized abdominal pain with a predominance of hypogastric pain that spread to the back and between the patient’s shoulders. The pain started suddenly and increased dramatically. The patient complained of no defecation, no gas-passing, and anorexia in the last four days. The pain was not feeding and positional related. The patient also complained of prolonged periods of constipation, which began eight years ago. He did not have a fever, nausea, or vomiting. The patient had stable vital signs at the time of admission. On abdominal examination, the patient had severe and progressive distension. He had severe and generalized tenderness in the abdomen with a predominance of epigastric, but rebound or guarding were not detected. His past medical history represented that the patient has had inflammatory bowel disease (IBD) since seven years ago. The rectal examination represented nothing. The patient was asked to do an upright chest X-ray and upright and supine abdominal X-ray. There was no free-air or free-fluid level in chest X-ray images, but abdominal X-ray showed obstruction view due to dilated bowel loops and air-fluid levels, as can be seen in Figures 1-3. 

**Figure 4:**
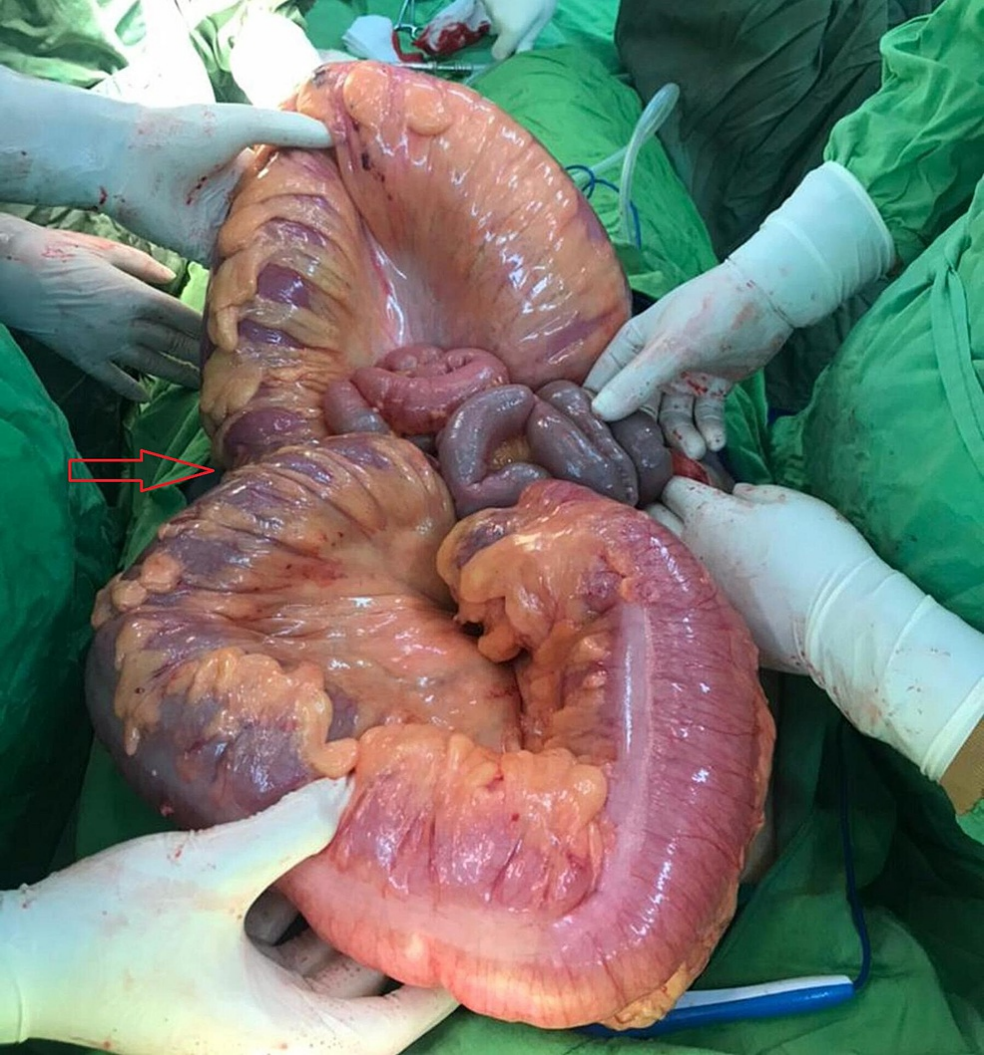
Transverse colon volvulus Red arrow shows the location of the volvulus

**Figure 5:**
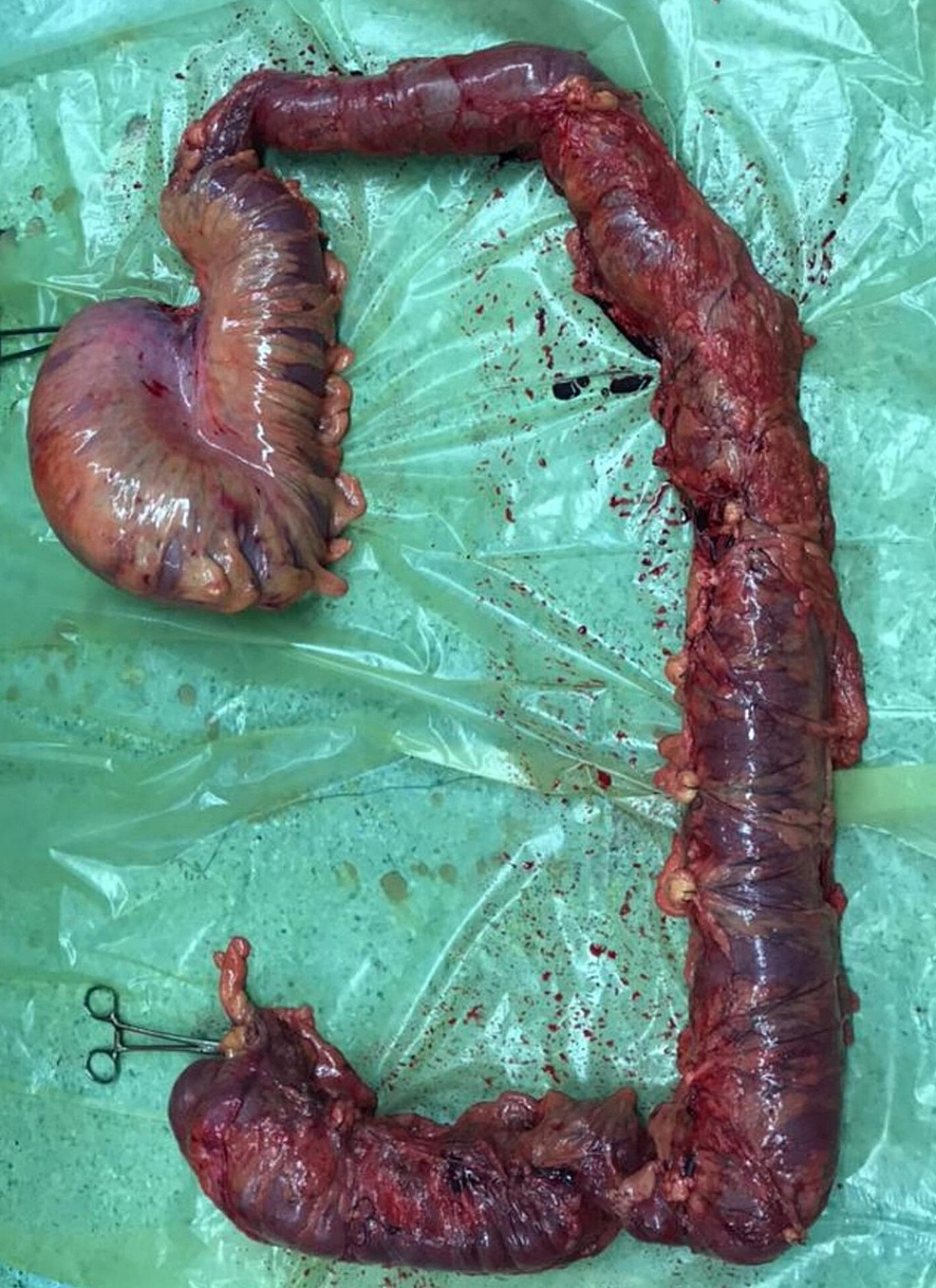
The colon after total colectomy

For further evaluation, the patient was asked to do a thoracoabdominal CT scan which confirmed dilated bowel loops and probability of obstruction and volvulus. The blood test analyzed presented a high level of erythrocyte sedimentation rate (ESR)=29 (usually should be under 15 in males) and lactate dehydrogenase (LDH) = 500 (normally should be under 280 in adults) and leukocytosis (white blood cells [WBC] = 11900 g/dL with a neutrophilia ratio of 83%) and metabolic acidosis, probably caused by decreased tissue perfusion. In continuance and due to suspicion of sigmoid volvulus and according to the clinical presentations and result of blood tests and imaging reports, the patient underwent laparotomy. After opening the patient’s abdomen, a severely distended colon was seen. The wall of the colon was thinner along its entire length, and it was ischemic. At first, the transverse colon volvulus was seen inconceivably, which was twisted around itself, as can be seen in Figure 4. So, this torsion was removed. After more exploration in the abdominal cavity, a second torsion was seen in the sigmoid part of the colon, in which the colon was twisted twice around itself. After the second torsion was removed, the patient underwent a total colectomy due to ischemia and necrosis tissue of the colon wall along its entire length, as can be seen in Figure 5. After ileorectal anastomosis, the abdomen was closed. The patient had a comfortable and proper recovery. After the operation, the patient was transferred to the ICU and was under care for one day there and transferred to the ward two days later. Proper and imperative treatments were started for him. The patient was discharged from the surgical service in good general condition after tolerating PO and defecation.

## Discussion

So far, few cases of simultaneous sigmoid and transverse colon volvulus in different people with and without specific past medical history and various ages have been reported as case reports [2,5]. So, Given the importance of the issue and the importance of paying attention to this disease, we have reported another case of it in this article. In this article, a case of simultaneous sigmoid colon volvulus and transverse colon volvulus was presented, which occurred in an old man with a past medical history of IBD for the last seven years. Intraoperatively, the transverse colon was mobile and twisted around itself, and the sigmoid colon was twisted twice around itself. In addition, the colon wall was thinner than usual, and there was evidence of ischemia along the entire length of the colon wall. As studies showed, the most common location of volvulus in the colon is the sigmoid part, with a probability of 40% to 60%. In comparison, the probability of occurrence of volvulus in the transverse colon is much lower and about 3% to 5%. Therefore, the possibility of simultaneous occurrence of these two events is very low and can be considered a rare phenomenon. The pathology of the disease and this phenomenon are not completely understood yet. Males are more at risk than females for this disease [1,6,7]. Several risk factors increase the probability of the occurrence of colonic volvuli, such as intestinal malrotation, an enlarged colon, a long mesentery, Hirschsprung disease, pregnancy, abdominal adhesions, and chronic constipation. Colonic volvulus is generally divided into three categories which are anatomical, physiological, and congenital. One of the anatomical risk factors in volvulus occurrence is inflammatory bowel disease which can be explained by the increased risk of intestinal dilation, torsion, and fixation in inflammatory bowel conditions [5,8]. This condition can change the patient’s defecation pattern, such as prolonged and chronic constipation [9]. As mentioned above, the patient has been suffering from IBD for seven years and prolonged periods of constipation for eight years. Since chronic constipation is one of the risk factors for colonic volvulus, it can be concluded that IBD such as Crohn's disease can be considered as a possible risk factor in these patients. Clinical examination is the first step in diagnosing this disease, but no particular symptoms and findings in the clinical examination could help the surgical team differentiate double volvulus, particularly from another part of the colon volvulus. Another important way that could be effective and useful is imaging, such as abdominal X-ray and CT scan, but choosing which one is more effective in the situation depends on the surgical team and the patient’s condition. In simultaneous volvulus conditions, diagnostic view and features such as coffee bean sign, north-ern exposure, or inverted U-shaped sign may not be seen [10]. However, as seen in our patient, diffuse colonic distention can be seen, which may require additional imaging investigation. The other imaging method is using a CT scan to illustrate two concomitant whirl signs [8,11]. Choosing between non-surgical or surgical treatment or even choosing which surgical technique is more effective and better for the patient depends on the patient’s condition and surgical team. In this case, the surgical team chose the laparotomy between surgical treatment methods according to the patient’s condition. Because of numerous ischemic areas and necrosis in the length of the colon wall, the surgical team decided to do a total colectomy and prepare an ileorectal anastomosis.

## Conclusions

However, one of the most important reasons for bowel obstruction is colon volvulus; simultaneous sigmoid and transverse colon volvulus is a rare condition that may occur in patients with obstruction bowel symptoms, and it can create emergency conditions for the patient and threaten the patient's life more than a single volvulus condition. Several clinical examination and imaging findings could help doctors evaluate and diagnose the patient’s condition and disease, but none are definitive. Choosing the best way between different surgical techniques depends on different things, especially the extent and severity of tissue necrosis. Because definitive and standard treatment protocol and definite criteria may not exist for patients with simultaneous volvulus and the patient's life may be highly endangered, careful examination of the patient and attention to the clinical examination and laboratory and imaging findings should be strictly and carefully considered.
